# Electrospun ZnO/Pd Nanofibers as Extremely Sensitive Material for Hydrogen Detection in Oxygen Free Gas Phase

**DOI:** 10.3390/polym14173481

**Published:** 2022-08-25

**Authors:** Vadim Platonov, Abulkosim Nasriddinov, Marina Rumyantseva

**Affiliations:** Chemistry Department, Moscow State University, 119991 Moscow, Russia

**Keywords:** electrospinning, nanofibers, zinc oxide, Pd clusters, semiconductor gas sensor, hydrogen, different oxygen backgrounds

## Abstract

The development of safety sensors is an urgent necessity for the successful use of hydrogen in real conditions, which may differ, in particular, by the oxygen content in the surrounding atmosphere. Palladium-modified zinc oxide shows the high sensitivity when detecting hydrogen in air; however, studies of the sensor properties and the operation mechanism of the ZnO/Pd sensor when reducing gases are detected in an oxygen deficient or inert atmosphere have not been effectuated. In this work, we synthesized the ZnO and ZnO/Pd nanofibers by electrospinning and for the first time determined their sensor properties in the detection of CO, NH_3_ and H_2_ in different oxygen backgrounds. The microstructure and composition of nanofibers were characterized by electron microscopy, X-ray diffraction, X-ray fluorescent spectroscopy, and X-ray photoelectron spectroscopy. The interaction with the gas phase was investigated in situ by diffuse reflectance IR Fourier transform spectroscopy (DRIFTS). The sensor properties of ZnO and ZnO/Pd nanofibers were studied at 100–450 °C towards CO, NH_3_ and H_2_ in the N_2_/O_2_ gas mixtures containing 0.0005–20% O_2_. When detecting CO, a decrease in the oxygen concentration from 20 to 0.0005% in the gas phase does not lead to a significant change in the sensor response. At the same time, when detecting NH_3_ and especially H_2_, a decrease in oxygen concentration down to 0.0005% results in the dramatic increase in the sensor response of ZnO/Pd nanofibers. This result is discussed in terms of palladium hydride formation, modulation of the potential barrier at the ZnO/Pd interface, as well as changes in the concentration of donor defects and charge carriers in the ZnO matrix. Synthesized electrospun ZnO/Pd nanofibers are extremely promising materials for sensors for detecting hydrogen in an oxygen free atmosphere.

## 1. Introduction

Hydrogen is a clean and renewable energy source, promising to replace carbon-containing fossil fuels, because it has high energy efficiency and zero carbon footprint. Recently, hydrogen has been widely used in advanced technologies related to the petroleum, chemical, aerospace industry, nuclear power, and in the development of fuel cells. Hydrogen has no color, odor or taste, is extremely flammable and is characterized by a high rate of flame propagation. Therefore, hydrogen leaks during its storage, transportation and use can lead to technogenic catastrophes. As the use of hydrogen is becoming more common in various industries, the detection of gaseous hydrogen using gas sensors is extremely important. 

The development of safety sensors is an urgent necessity for the successful use of hydrogen in real conditions, which may differ, in particular, by the oxygen content in the surrounding atmosphere. Semiconductor gas sensors based on metal oxides SnO_2_, ZnO, In_2_O_3_, etc., have high sensitivity to hydrogen not only in air, but also in inert atmosphere [1]. Recently, a large number of papers have been published [2,3,4,5,6,7,8,9,10,11,12,13,14,15,16,17,18,19], which show the high 45–51 sensitivity of palladium-modified zinc oxide when detecting hydrogen in air. It is assumed that the role of palladium lies in the synergistic effect of electronic and chemical sensitization, as well as in the ability to dissolve hydrogen gas. At the same time, studies on the mechanism of ZnO/Pd sensor response during detection of reducing gases, in particular hydrogen, in oxygen deficient or inert atmosphere have not been carried out. This kind of experimental information and theoretical modeling are presented only for SnO_2_ based materials [20,21,22,23,24]. 

Over the past decade, nanofibers of different natures have been endowed with “smart” properties to enable a variety of new applications [25,26,27,28,29]. One-dimensional (1D) nanostructured semiconductor oxides in the form of nanofibers attract special attention as materials for gas sensors due to the large length-to-diameter ratio and high surface-to-volume ratio, which can lead to increased sensitivity [26,27,28,29,30,31,32,33,34,35]. Electrospinning offers a relatively simple and versatile method for generating 1D nanostructures (nanofibers) that are exceptionally long in length, uniform in diameter, and diverse in composition [25,30,31,32]. 

In this work, we investigated the gas sensor properties of electrospun ZnO and ZnO/Pd nanofibers in the detection of CO, NH_3_ and H_2_ in different oxygen backgrounds. The composition and microstructure parameters of the synthesized electrospun nanofibers were carefully characterized by electron microscopy, X-ray diffraction (XRD), X-ray fluorescent spectroscopy (XRF), and X-ray photoelectron spectroscopy (XPS). The interaction with the gas phase was investigated in situ by diffuse reflectance IR Fourier transform spectroscopy (DRIFTS). 

## 2. Materials and Methods

### 2.1. Materials Synthesis

The polymer solution was prepared according to the following scheme.

A zinc acetate Zn(CH_3_COO)_2_·2H_2_O (Sigma-Aldrich, >99%, St. Louis, MO, USA) precursor was added to 5 mL of 2-methoxyethanol (Sigma-Aldrich, 99.8%, St. Louis, MO, USA) and stirred with a magnetic stirrer at 40 °C until completely dissolved (solution “A”). Polyvinylpyrrolidone (Sigma-Aldrich, >99%, St. Louis, MO, USA) (PVP, molecular weight 1,300,000, 900 mg) was added to 5 mL of isopropyl alcohol (Sigma-Aldrich, >99.9%, St. Louis, MO, USA) (IPA) and stirred at 40 °C until completely dissolved (solution “B”). Then, solution "A" was slowly (drop by drop) added to solution "B" under continuous stirring. The resulting solution was additionally stirred for 1 h. The precursor solution was placed in a medical syringe fixed in a syringe pump Beyond BYZ-810 (Changsha Beyond Medicial Devices Co., Ltd., Changsha, Hunan Province, China), and at a rate of 1 mL/h was fed to a metal needle with an internal diameter of 510 μm. The collector, made in the form of a stainless wire frame, was located at a distance of 125 mm from the needle. The voltage between the needle and the collector was maintained at 10–11 kV by a high voltage source. The resulting polymer nanofibers were collected and annealed in air at 550 °C for 5 h (heating rate of 1K/min). The optimal annealing regime was determined based on the data of thermogravimetric analysis with mass-spectral determination of gaseous products (TG–MS) on the NETZSCH STA 409 PC/PG (NETZSCH, Selb, Germany) analyzer [36]. Palladium modified ZnO/Pd nanofibers were obtained by a similar method, after adding palladium precursor (palladium acetylacetonate Pd(acac)_2_, Pd(CH_3_COCHCOCH_3_)_2_) (Sigma-Aldrich, >99%, St. Louis, MO, USA) to solution “A”.

### 2.2. Materials Characterization

The microstructure of the nanofibers was investigated using scanning electron microscopy (SEM) on a Carl Zeiss NVision 40 (Carl Zeiss, Inc., Oberkochen, Germany) microscope with an intra-lens detector at an accelerating voltage of 5 kV. The phase composition and the crystallite size of the annealed nanofibers were determined by X-ray diffraction (XRD) on the DRON–4M instrument (Cu K_α_ radiation, wavelength 1.54051 Å) (Burevestnik, St. Petersburg, Russia). The crystallite size values (*d_XRD_*) for ZnO phase were estimated from the broadening of the most intense wurtzite reflections (100), (002), and (101) using the Scherrer formula. The specific surface area (S_BET_) was calculated using BET (Brunauer–Emmett–Teller) model from nitrogen adsorption isotherms obtained on ASAP 2010 instrument (Micromeritics, Norcross, GA, USA). Before the measurements, the samples were evacuated at 300 °C to 4 × 10^−1^ Pa for 3 h. 

The palladium content in ZnO/Pd nanofibers (1 wt% Pd) was confirmed by X-ray fluorescence (XRF) using the M1 Mistral micro-X-ray spectrometer (Bruker, Billerica, MA, USA). The element’s distribution in ZnO/Pd nanofibers was investigated using high angle annular dark field scanning transmission electron microscopy (HAADF-STEM), and energy-dispersive X-ray mapping (STEM-EDX) on a FEI Osiris microscope with a Super-X detector at 200 kV.

The surface composition of nanofibers and the chemical state of the elements (Zn, O, Pd) were studied by X-ray photoelectron spectroscopy (XPS) using an Axis Ultra DLD spectrometer (Kratos, Manchester, UK). The spectra were obtained using monochromated Al K_α_ radiation. The charge shift was corrected using C 1s ground state peak with a binding energy of 285.0 eV. The spectra for the Zn 2p, O 1s and Pd 3d regions were recorded with a step of 0.1 eV. The spectra were fitted by mixed Gaussian–Lorentz functions with the background calculation by the Shirley method.

The sensor properties of nanofibers were studied in situ by measuring the electrical conductivity of thick films. The ZnO and ZnO/Pd nanofibers were mixed with α-terpineol to form a paste, which was deposited onto alumina microhotplates 2 × 0.5 × 0.1 mm with Pt electrodes (the distance between the electrodes is 0.1 mm) and embedded Pt-meander serving as a heater. After deposition, the paste was dried at 50 °C for 5 h, heated at a rate of 2 K/min to 450 °C and kept at this temperature for 24 h to completely remove the binder. The temperature was controlled by the resistance of the platinum heater. The sensor resistance was measured at 1.3 V DC-voltage under controlled gas flow of 100 ± 0.1 mL/min at a temperature fixed in the range of 100–550 °C with the step of 50 °C. The sensor parameters of ZnO and ZnO/Pd nanofibers to CO, NH_3_ and H_2_ reducing gases were studied in dry conditions (relative humidity at 25 °C RH = 0%) with different oxygen backgrounds (C(O_2_) = 20%, 15%, 10%, 5% and 0.0005%). The oxygen concentration was varied by diluting pure dry air (80% nitrogen, 20% oxygen) with high purity nitrogen (containing 0.0005% oxygen). Certified gas mixtures containing CO (5110 ppm), NH_3_ (2590 ppm) or H_2_ (1030 ppm) in nitrogen were used as gas sources. To maintain the oxygen level of 20% in gas mixtures containing a preassigned concentration of reducing gas, pure oxygen was additionally added. All gas mixtures were created using electronic flow meters EL–FLOW (Bronkhorst). The sensor response *S* was calculated as

(1) $$S=\frac{G_{gas}-G_{0}}{G_{0}}=\frac{R_{0}}{R}-1$$

where $G_{0}=\frac{1}{R_{0}}$ is the sample’s conductance in background gas with preassigned oxygen concentration, $G_{gas}=\frac{1}{R_{gas}}$ is the sample’s conductance in a gas mixture containing reducing gas CO, NH_3_ or H_2_. 

The interaction of ZnO/Pd nanofibers with CO, NH_3_ and H_2_ in dry air (20% oxygen) and dry nitrogen (0.0005% oxygen) was studied using the Diffuse Reflectance IR Fourier spectroscopy (DRIFTS) on a Perkin Elmer Frontier spectrometer (Perkin Elmer Inc., Beaconsfield, UK) with DiffusIR annex and HC900 flow chamber (Pike Technologies, Madison, WI, USA). Spectra were recorded in the 4000–1000 cm^−1^ range with 1 cm^−1^ step and accumulation of 30 scans.

## 3. Results and Discussion

**Figure 1:**
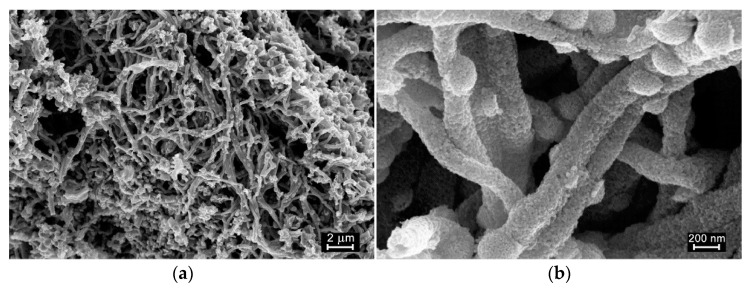
SEM images of ZnO nanofibers at different magnifications: (**a**) 10,000× and (**b**) 100,000×.

**Figure 2:**
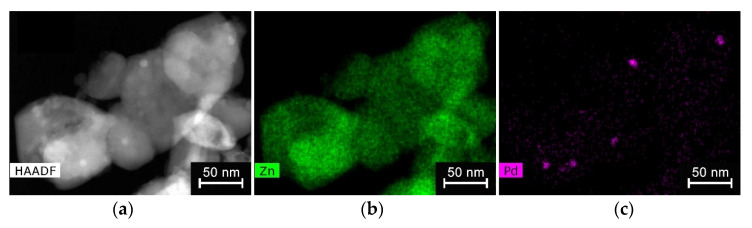
HAADF-STEM image (**a**) and STEM-EDX maps of Zn (**b**) and Pd (**c**) of ZnO/Pd sample.

The microstructure parameters and composition of ZnO and ZnO/Pd nanofibers are described in detail in our previous article [36] and summarized in Table 1. To illustrate, the fibrous structure of the electrospun material and the distribution of palladium clusters are shown in SEM images (Figure 1) and STEM-EDX maps (Figure 2). The electrospun fibers obtained after the polymer removal are formed by aggregated nanoparticles of zinc oxide with a wurtzite structure. Reflections of palladium-containing phases do not appear on diffractograms because of the low palladium content. According to XPS, palladium in nanofibers is present in the oxidized state Pd(II). The introduction of palladium does not lead to a significant change in the microstructure parameters of nanofibers; however, it causes a significant increase in the nanofiber’s resistance and a decrease in the concentration of surface oxygen-containing species (manifested by reducing the O_ads_/O_lat_ ratio of the O1s XP-spectrum components, where O_lat_ corresponds to the oxygen anions of the ZnO crystal structure, and O_ads_ corresponds to surface oxygen species). Both results seem to be due to the formation of a *p*-PdO/*n*-ZnO heterocontact and the transfer of electrons from an oxide with a smaller electron work function (φ = 4.6 eV, ZnO) to an oxide with a greater one (φ = 6.04 eV, PdO).

**Figure 3:**
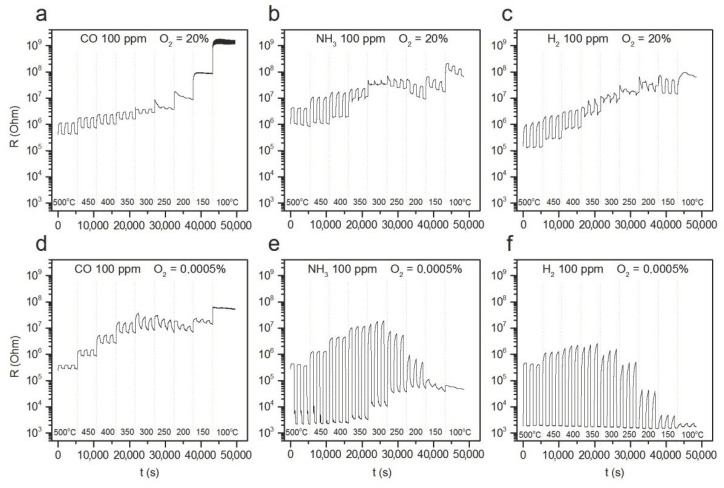
Transient sensor response of ZnO/Pd nanofibers to 100 ppm of reducing gases CO (**a**,**d**), NH_3_ (**b**,**e**) and H_2_ (**c**,**f**) in dry atmosphere (relative humidity at 25 °C RH = 0%) with different oxygen backgrounds of 20% (**a**–**c**) and 0.0005% (**d**–**f**) in the temperature range 100–500 °C.

**Figure 4:**
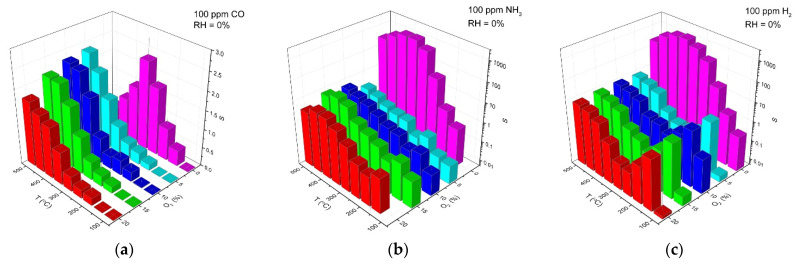
Temperature dependences of sensor response of ZnO/Pd nanofibers to 100 ppm of reducing gases CO (**a**), NH_3_ (**b**) and H_2_ (**c**) in dry atmosphere (RH = 0%) with different oxygen backgrounds C(O_2_) = 20, 15, 10, 5 and 0.0005%.

Figure 3 shows the resistance change of ZnO/Pd nanofibers with a cyclic variation in the gas phase composition during detection of 100 ppm of reducing gases CO, NH_3_ and H_2_ in atmosphere with different oxygen backgrounds of 20% and 0.0005% O_2_ in the temperature range 100–500 °C. The temperature dependences of the sensor response calculated by equation (1) are shown in Figure 4. Similar data for ZnO nanofibers are shown in Figures S1 and S2 in Supplementary Information.

**Figure 5:**
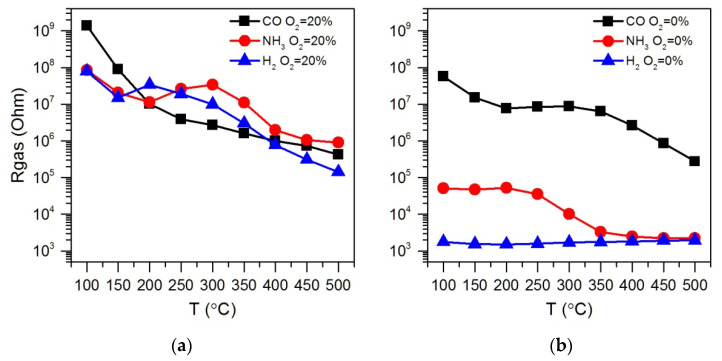
Temperature dependences of ZnO/Pd resistance in the presence of 100 ppm of CO, NH_3_ or H_2_ in dry atmosphere (RH = 0%) with different oxygen backgrounds 20% O_2_ (**a**) and 0.0005% O_2_ (**b**).

**Figure 6:**
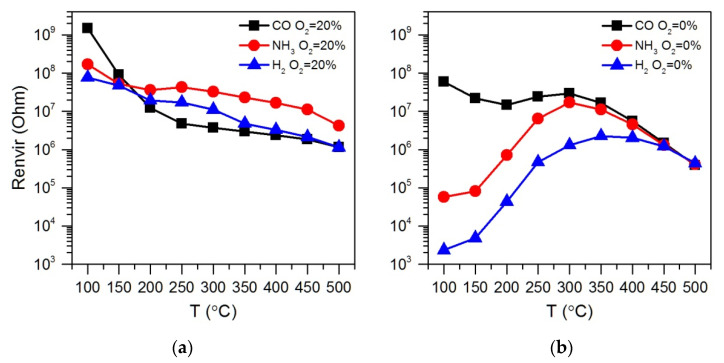
Temperature dependences of ZnO/Pd resistance in dry atmosphere (RH = 0%) with different oxygen backgrounds 20% O_2_ (**a**) and 0.0005% O_2_ (**b**) after interaction with 100 ppm of reducing gases CO, NH_3_ or H_2_.

The obtained results allow us to identify the following patterns:

(i) The sensor response of ZnO and ZnO/Pd nanofibers when detecting CO, NH_3_ and H_2_ weakly depends on the oxygen content in the background gas in the concentration range of 20–5% O_2_.
(ii) Lowering the oxygen concentration down to 0.0005% does not significantly affect the magnitude of the sensor response of ZnO and ZnO/Pd nanofibers toward CO. However, when NH_3_ and H_2_ are detected, the sensor response of ZnO/Pd nanofibers increases by three orders of magnitude.
(iii) Such a sharp increase in the sensor response is mainly determined by a sharp decrease in the resistance of ZnO/Pd nanofibers in the presence of 100 ppm ammonia and hydrogen. Moreover, the temperature intervals for such a decrease in resistance differ significantly for these gases: 350–500 °C in NH_3_ and 100–500 °C in H_2_ (Figure 5).
(iv) The resistance of ZnO/Pd nanofibers in the background gas containing 20% O_2_, which is achieved within 15 min after interaction with 100 ppm of reducing gases CO, NH_3_ or H_2_, turned out to be quite close in the entire temperature range (Figure 6a). However, in the background gas containing 0.0005% O_2_ (Figure 6b), the resistance values after interaction with NH_3_ and H_2_ in the low temperature range of 100–300 °C are several orders of magnitude less than in the case of CO.

Thus, the main differences in the sensor properties of ZnO and ZnO/Pd nanofibers are that in the air (20% O_2_) and in the absence of oxygen (0.0005% O_2_), the magnitude of the sensor response of unmodified ZnO during the NH_3_ and H_2_ detection practically does not change, while the sensor response of ZnO/Pd in oxygen free background gas increases dramatically. To explain the observed trends, we will consider the main processes occurring on the surface of ZnO and ZnO/Pd when interacting with reducing gases in the background atmosphere with different oxygen concentrations. 

It is well established that the formation of the sensor response of *n*-type semiconductor oxides during the detection of reducing gases in dry air (20% O_2_) occurs with the participation of oxygen chemisorbed on the surface of the oxide ($O_{\mathrm{ads}}^{-\alpha }$ in reaction (2)), and/or lattice oxygen anions in the near-surface layer ($O_{\mathrm{lat}}$ in reaction (3)):

(2) $${\mathrm{Red}}_{\mathrm{gas}}+O_{\mathrm{ads}}^{-\alpha }\to {\mathrm{RedO}}_{\mathrm{gas}}+{\alpha e}^{-}$$

(3) $${\mathrm{Red}}_{\mathrm{gas}}+O_{\mathrm{lat}}\to {\mathrm{RedO}}_{\mathrm{gas}}+V_{O}^{2+}+2e^{-}$$

where Red denotes the molecule of reducing gas, and RedO is its oxidized form.

Both processes lead to an increase in the concentration of the main charge carriers (electrons) in the conduction band of the semiconductor and an increase in electrical conductivity (a decrease in resistance). In the case of SnO_2_, the main process is the oxidation of reducing gases with chemisorbed oxygen (reaction (2)), and in the case of ZnO and WO_3_, the oxidation of reducing gases by the Mars–van Krevelen mechanism (reaction (3)) makes a significant contribution to the formation of a sensor response [36,37,38]. 

The authors [22] demonstrated by the example of SnO_2_ that for the case of H_2_, in addition to interaction with oxygen ions, there is also a process of formation of surface donors (rooted hydroxyl groups), which explains the sensor effect in the absence of oxygen. When there is a sufficient amount of oxygen ions on the surface (a large concentration of oxygen in the background gas), the sensor response is due to the formation and change of the electron depletion layer, which is determined by the concentration of surface acceptors. When they are absent (very low oxygen concentration in the background gas), surface donors come into play, and the conduction mechanism changes as the electron accumulation layer forms on the surface. In the case of CO, there is no clear candidate for a type of CO-related surface species that could play the role of a donor [23]; however, the CO molecule, whose electronic structure determines the combination of σ-donor (the presence of an electron pair on the binding 3σ-orbital) and π-acceptor properties, is adsorbed on coordinatively unsaturated Sn_5C_^4+^ cations (5C denotes coordination number 5 instead of 6, which corresponding for Sn^4+^ in SnO_2_ crystal structure) with an energy of 0.25 eV [39]. Experiments on degassing in vacuum have shown a fairly stable binding of Sn_5C_^4+^–CO [39], despite the low value of the adsorption energy. This type of CO–metal oxide surface binding implies the transfer of electron density from CO to the adsorbent, i.e., the semiconductor oxide. The ammonia molecule has a significantly higher dipole moment equal to 4.48 D compared to CO (0.11 D). The presence of a free sp^3^-hybridized electron pair determines the pronounced donor properties of NH_3_. Ammonia is adsorbed on the surface of semiconductor oxides to form bonds with Lewis acid sites–coordinatively unsaturated metal cations [38,40]. Thus, the idea of the formation of donor particles and an electron accumulation layer can also be applied to explain the formation of the sensor response of semiconductor oxides in NH_3_ detection in an oxygen-deficient background gas.

In [22,23], a decrease in the signal of the SnO_2_ sensor to H_2_ and CO with an increase in the oxygen concentration in the background gas was demonstrated. The authors explained the observed effect by electron capture by oxygen acceptors/ions present on the surface. In the case of ZnO nanofibers, an increase in the sensor response to CO and NH_3_ with a decrease in the oxygen concentration in the background gas down to 0.0005% O_2_ was not detected (Figure S2, Supplementary Information). This may be due to the dominant contribution of reaction (3), which is not associated with the participation of surface acceptors–chemisorbed oxygen species, to the sensor response.

The effect of palladium on the sensor properties of semiconductor oxides when detecting reducing gases in air (20% O_2_) is realized through a combination of chemical and electronic sensitization. Chemical effects include oxygen and hydrogen spillover and specific adsorption of the detected gases on the surface of Pd clusters. The specific adsorption of CO molecules on Pd^0^ is accompanied by a weakening of the intramolecular C–O bond and a decrease in the activation energy of further transformations [41].

The electronic mechanism consists in partial reduction and an increase in the proportion of Pd^0^ due to oxidation of reducing gas molecules on the surface of PdO_x_ clusters, for example:

(4) $${\mathrm{CO}}_{\mathrm{gas}}+{\mathrm{PdO}}_{x}\to {\mathrm{CO}}_{2\;\mathrm{gas}}+\mathrm{Pd}$$

The Pd^0^ work function (φ = 4.8 eV) is less than in that of PdO_x_ (φ = 6.04 eV). Thus, as the result of the reaction (4), the barrier at the ZnO/Pd interface is removed, which leads to a decrease in sample resistance. This effect ensures the preservation of ZnO/Pd sensitivity when detecting CO at high humidity (relative humidity at 25 °C RH = 60%), while blank ZnO nanofibers completely lose sensitivity under these conditions [36]. A large number of works devoted to the sensor properties of ZnO/Pd when detecting hydrogen in air consider the processes of PdO_x_ to Pd^0^ reduction during hydrogen oxidation to H_2_O, and the corresponding change in the barrier at the ZnO/Pd interface. The process of detecting ammonia is complex and is often characterized by a temperature dependence of the sensor response, which has a minimum in the average temperature region (Figure 6b). This is because of an abnormal resistance behavior (see, for example, Figure 3b, T = 300 °C) that may be due to the catalytic transformation of NH_3_ into NO_x_ over PdO_x_ clusters with subsequent adsorption of electron acceptor species on semiconductor oxide surface [42,43]. 

Comparing the results presented in Figure 3a,c and the temperature dependences of the ZnO/Pd sensor response when detecting CO in a background gas containing 20% O_2_ and 0.0005% O_2_ (Figure 4a), we can only note a shift of the maximum sensor response to the lower temperature region with a decrease in oxygen concentration down to 0.0005% O_2_. This indicates the preservation of the mechanism of sensor response formation, determined, apparently, by the reduction of PdO_x_ clusters via reaction (4), which, in the absence of oxygen, occurs at a lower temperature.

The similarity of the results obtained when detecting H_2_ and NH_3_ in an oxygen-deficient atmosphere of 0.0005% O_2_ leads to the assumption that the same mechanism for detecting these gases is implemented, but radically different from the case of CO. It is well known that palladium is capable of absorbing hydrogen to form PdH_x_ hydrides. Hydrogen absorption in Pd occurs by a two-stage mechanism with dissociation and adsorption of a hydrogen molecule on the metal surface as the first stage [44]. The palladium–hydrogen phase diagram [45] indicates the occurrence of two phases, α-PdH_x_, (x_max_ = 0.015) and β-PdH_x_ (x_min_ = 0.607) (reaction (5)). The formation of PdH_x_ results in the change in the electron work function and can contribute to the transfer of electrons in the system, which leads to the modulation of the electrical resistance.

(5) $$\frac{x}{2}H_{2\;\mathrm{gas}}+\mathrm{Pd}↔{\mathrm{PdH}}_{x}$$

**Figure 7:**
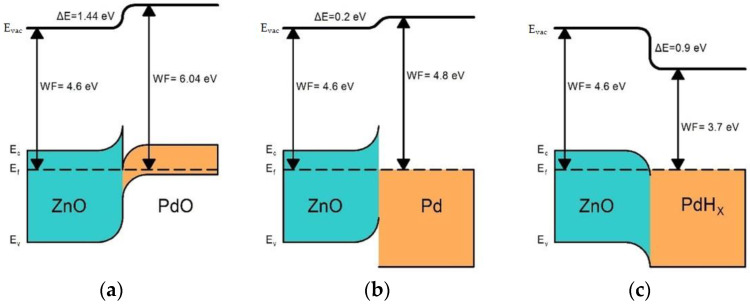
Energy diagrams of heterocontacts formed at the ZnO/PdO (**a**), ZnO/Pd (**b**), and ZnO/PdH_x_ (**c**) interfaces. E_C_—conduction band; E_V_—valence band; E_f_—Fermi level; E_vac_—vacuum level; WF—work function.

Thus, the formation of the ZnO/Pd sensor response when detecting hydrogen in an oxygen-deficient atmosphere can be represented as follows (Figure 7). The reduction of PdO_x_ clusters (φ = 6.04 eV) to metallic Pd^0^ (φ = 4.8 eV) leads to a decrease in the barrier at the ZnO/Pd boundary; however, electron transfer from ZnO (φ = 4.6 eV) to Pd still occurs, which implies the preservation of a high level of resistance in ZnO/Pd nanofibers. The dissociation and absorption of hydrogen by Pd^0^ clusters with formation of a PdH_x_ with work function φ = 3.7 eV [46] results in the destruction of the Schottky barrier on the PdH_x_/ZnO interface and the formation of the electron accumulation layer on the ZnO surface. This leads to a dramatic decrease in the resistance of ZnO/Pd nanofibers, providing an exceptionally great sensor response. 

**Figure 8:**
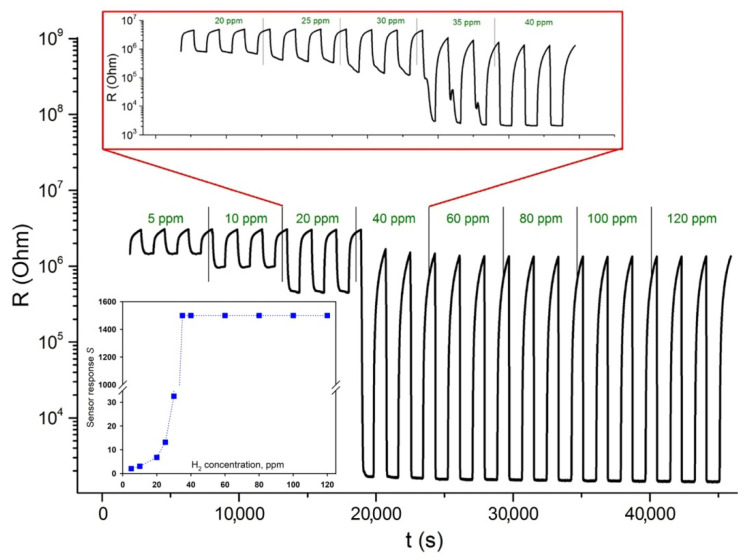
Transient sensor response of ZnO/Pd nanofibers to 5–120 ppm H_2_ in background gas with 0.0005% O_2_ at 350 °C. Inset: Sensor response S as a function of H_2_ concentration.

It is obvious that for the formation of such an accumulation layer on the ZnO surface, a complete transformation of Pd^0^ into PdH_x_ is necessary. Figure 8 demonstrates the resistance change of ZnO/Pd nanofibers with a cyclic variation in the gas phase composition during detection of 5–120 ppm H_2_ in background gas with 0.0005% O_2_ at 350 °C. It can be seen that such a complete transformation (at 350 °C) becomes possible at H_2_ concentration of 35 ppm or more. In these cases, there is a change in resistance by three orders of magnitude, and the resistance in the presence of hydrogen (and, consequently, sensor response) is already independent of the H_2_ concentration in the gas phase.

The similarity of the results obtained during the detection of hydrogen and ammonia may be due to NH_3_ decomposition (reaction (6)) on the ZnO surface in the absence of oxygen at high temperatures with the formation of nitrogen and hydrogen, which, in turn, undergoes spillover on reduced palladium clusters and forms PdH_x_.

(6) $$2{\mathrm{NH}}_{3\;\mathrm{gas}}↔N_{2\;\mathrm{gas}}+3H_{2\;\mathrm{gas}}$$

The formation of ammonia is exothermic, therefore, with an increase in the measurement temperature, the equilibrium (6) shifts to the right, providing a higher concentration of hydrogen in the gas phase. Figure 5b shows that at a temperature of 350 °C and above, the resistance achieved in a background gas with 0.0005% O_2_ in the presence of 100 ppm NH_3_ coincides with the resistance achieved in the presence of 100 ppm H_2_. This indicates that in this temperature range, the concentration of hydrogen formed due to NH_3_ decomposition is sufficient for the formation of PdH_x_ and an accumulation layer on the ZnO surface.

The return of the resistance of the ZnO/Pd nanofibers to the initial value when hydrogen is removed from the background gas containing 0.0005% O_2_ is due to the decomposition of PdH_x_. From the kinetic investigations made in [44], it was concluded that at low oxygen concentrations (e.g., below 1% O_2_), the hydrogen removal is dominated by hydrogen recombination. With an increase in the partial pressure of oxygen in the background gas, the removal of hydrogen from the PdH_x_ accelerates, and the formation of water becomes the dominant reaction. Despite the fact that the authors of [46] postulated the formation of PdH_x_ even when detecting H_2_ in air, this conclusion seems to be debatable, since in the presence of a high concentration of oxygen, the process of oxidation of H_2_ with the formation of H_2_O is more likely. It should also be noted that in this case, the change in resistance and the corresponding values of the sensor response were not so significant.

**Figure 9:**
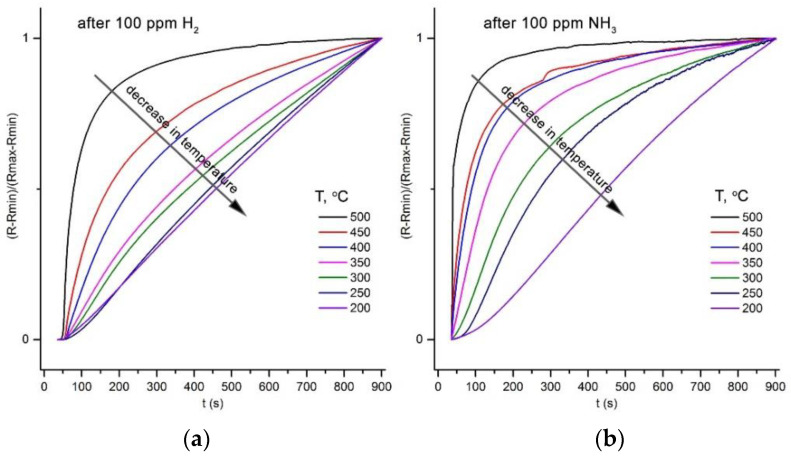
Temperature dependence of the rate of the resistance recovery in background gas with 0.0005% O_2_ after 100 ppm H_2_ (**a**) or 100 ppm NH_3_ (**b**) is turned off.

The PdH has the negative enthalpy of formation [4], therefore, an increase in temperature leads to a shift of the equilibrium (5) to the left and an increase in the rate of decomposition of palladium hydride into simple substances. On the contrary, at low temperatures, the rate of the hydrogen removal is insufficient to the resistance of the ZnO/Pd nanofibers, which returns to the initial value when hydrogen is removed from the background gas during the recovery part of the sensor measurement cycle (15 min, Figure 3f and Figure 6b). This assumption is confirmed by a decrease in the rate of increase of the normalized resistance value (R − R_min_)/(R_max_ − R_min_), where R is the resistance at a given time, R_min_ is the minimum resistance in hydrogen, and R_max_ is the maximum resistance that is reached 15 min after the hydrogen is turned off (Figure 9a). A similar decrease in the rate of resistance recovery is observed after interaction with NH_3_ (Figure 9b). However, in this case, a decrease in temperature also leads to a decrease in the concentration of hydrogen in the gas phase and a decrease in the amount of hydrogen absorbed by palladium clusters. As a result, achieving higher absolute resistance values in the background gas 15 min after NH_3_ is turned off becomes possible (Figure 3e and Figure 6b).

**Figure 10:**
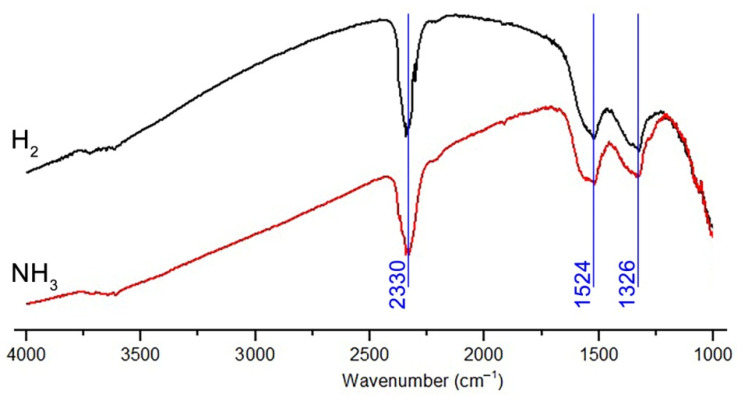
DRIFT spectra of ZnO/Pd nanofibers recorded after 120 min of exposure in the presence of 500 ppm H_2_ and 500 ppm NH_3_ in background gas with 0.0005% O_2_ at 350 °C.

To verify the conclusion about the same mechanism of formation of the ZnO/Pd sensor response when detecting H_2_ and NH_3_ in the absence of oxygen, the study of the interaction of ZnO/Pd nanofibers with 500 ppm H_2_ and 500 ppm NH_3_ in background gas with 0.0005% O_2_ was carried out in situ by DRIFTS method. The full set of the obtained spectra is presented on Figure S3 (Supplementary Information). The spectra recorded after 120 min of exposure in the presence of 500 ppm H_2_ and 500 ppm NH_3_ at 350 °C are shown in Figure 10. Spectrum in pure nitrogen (0.0005% O_2_) was used as the baseline. An intense negative band at 2330 cm^−1^ indicates desorption of CO_2_ molecules. As discussed previously [36], the nature of the two negative bands at 1524 and 1326 cm^−1^ is not clear [47,48]. It was demonstrated that the positive absorption intensity in this region increases with the increase in the partial pressure of oxygen in the surrounding atmosphere [49], while the negative intensity is observed in the presence of reducing gases CO and H_2_ [47] and increases with the increase in the heating time in high vacuum at T = 370 °C [49]. It can be assumed that together with the observed modulation of the spectrum base line indicating the increasing charge density in the semiconductor oxide [49], the occurrence of these bands corresponds to the formation of an accumulating layer on the ZnO surface. Since there are no qualitative differences in the spectra obtained in the presence of NH_3_ and H_2_, it can be assumed that the formation of the ZnO/Pd sensor response when detecting H_2_ and NH_3_ in the absence of oxygen occurs according to the same scenario, including the following processes: (i) reduction of PdOx clusters to Pd^0^; (ii) spillover of hydrogen (introduced into the gas phase or formed as a result of NH_3_ decomposition) and its adsorption on the surface of the reduced Pd^0^ clusters; (iii) formation of palladium hydrides PdH_x_, characterized by lower electron work function compared to ZnO; (iv) formation of electron accumulation layer on the ZnO surface as a result of electron transfer from PdH_x_. The presence of oxygen in the background gas at a concentration of 1% or more [44], the formation of palladium hydride turns out to be impossible, since hydrogen is oxidized by oxygen on the surface of PdO_x_ clusters to form H_2_O molecules.

## 4. Conclusions

The sensor properties of electrospun ZnO/Pd nanofibers were studied at 100–450 °C towards CO, NH_3_ and H_2_ in the N_2_/O_2_ gas mixtures containing 0.0005–20% O_2_. It was found that lowering the oxygen concentration down to 0.0005% does not significantly affect the magnitude of the sensor response of ZnO and ZnO/Pd nanofibers toward CO. However, when NH_3_ and H_2_ are detected in the absence of oxygen, the sensor response of ZnO nanofibers practically does not change compared to that obtained at higher oxygen concentrations, while the sensor response of ZnO/Pd nanofibers increases by three orders of magnitude. The results obtained when detecting H_2_ and NH_3_ in an oxygen-deficient atmosphere of 0.0005% O_2_ leads to the assumption that the same mechanism for detecting these gases is implemented, but radically different from the case of CO. For ZnO/Pd nanofibers, the common scenario includes reduction of PdO_x_ clusters to Pd^0^; spillover of hydrogen (introduced into the gas phase or formed as a result of NH_3_ decomposition) and its adsorption on the surface of the reduced Pd^0^ clusters; formation of palladium hydrides PdH_x_, characterized by lower electron work function compared to ZnO; and formation of an electron accumulation layer on the ZnO surface as a result of electron transfer from PdH_x_. The qualitative identity of the spectra obtained in situ at ZnO/Pd exposures of 500 ppm H_2_ and 500 ppm NH_3_ in oxygen-deficient background gas confirms this conclusion. It is important to note that this scenario can be realized only at an exceptionally low oxygen concentration in the background gas (in our case 0.0005%). At higher oxygen concentrations in the background gas (1% or more), the formation of palladium hydrides is impossible, since hydrogen is oxidized by oxygen on the surface of PdO_x_ clusters to form H_2_O molecules.

## Figures and Tables

**Table 1:** Summary of ZnO and ZnO/Pd nanofibers characteristics [36].

Sample	Fibers Diameter nm ^(1)^	Phase Composition ^(2)^	Palladium			ZnO Particle Size nm		S_BET_ m^2^/g	O_ads_/O_lat_^(6)^	R Ohm ^(7)^
			Content wt% ^(3)^	Oxidation State ^(4)^	Cluster Size nm ^(5)^	d_XRD_^(2)^	d_SEM_^(1)^			
ZnO	200–500	wurtzite	-	-	-	13 ± 1	20–40	10 ± 1	0.90	3 × 10^5^
ZnO/Pd	150–200	wurtzite	1.06 ± 0.07	Pd(II)	5–14	13 ± 1	20–40	12 ± 1	0.74	2 × 10^7^

^(1)^ From SEM; ^(2)^ from XRD; ^(3)^ from XRF; ^(4)^ from XPS; ^(5)^ from STEM-EDX maps; ^(6)^ area ratio of the O 1s XP-spectrum components; ^(7)^ resistance in dry air at 350 °C.

## Data Availability

The data presented in this study are available on request from the corresponding author.

## Supplementary Materials

The following supporting information can be downloaded at: https://www.mdpi.com/article/10.3390/polym14173481/s1, Figure S1: Transient sensor response of ZnO and ZnO/Pd nanofibers to 100 ppm of reducing gases CO (a,c), NH_3_ (b,d) in atmosphere with different oxygen backgrounds of 20% (a,b) and 0.0005% (c,d) in the temperature range 100–500 °C; Figure S2: Temperature dependences of sensor response of ZnO nanofibers to 100 ppm of reducing gases CO (a), NH_3_ (b) in atmosphere with different oxygen backgrounds C(O_2_) = 20, 15, 10, 5 and 0.0005%; Figure S3: DRIFT spectra of ZnO/Pd nanofibers recorded in the presence of 500 ppm H_2_ (a) and 500 ppm NH_3_ (b) in background gas with 0.0005% O_2_ at 350 °C.
