# Supplementary material for: Electrospun ZnO/Pd Nanofibers as Extremely Sensitive Material for Hydrogen Detection in Oxygen Free Gas Phase
Source: Polymers (Basel). 2022 Aug 25;14(17):3481. doi: 10.3390/polym14173481 (PMC9459723; doi:10.3390/polym14173481)
Supplement: Supplementary file 1 [file polymers-14-03481-s001.zip › polymers_Supplementary Information.pdf]

Electrospun ZnO/Pd Nanofibers as Extremely Sensitive Material for Hydrogen Detection in Oxygen Free Gas Phase

Vadim Platonov, Abulkosim Nasriddinov and Marina Rumyantseva *

Chemistry Department, Moscow State University, Moscow, 119991 Russia

* Correspondence: roum@inorg.chem.msu.ru; Tel.: +7-495-939-5471 (M.R.)

Supplementary Information

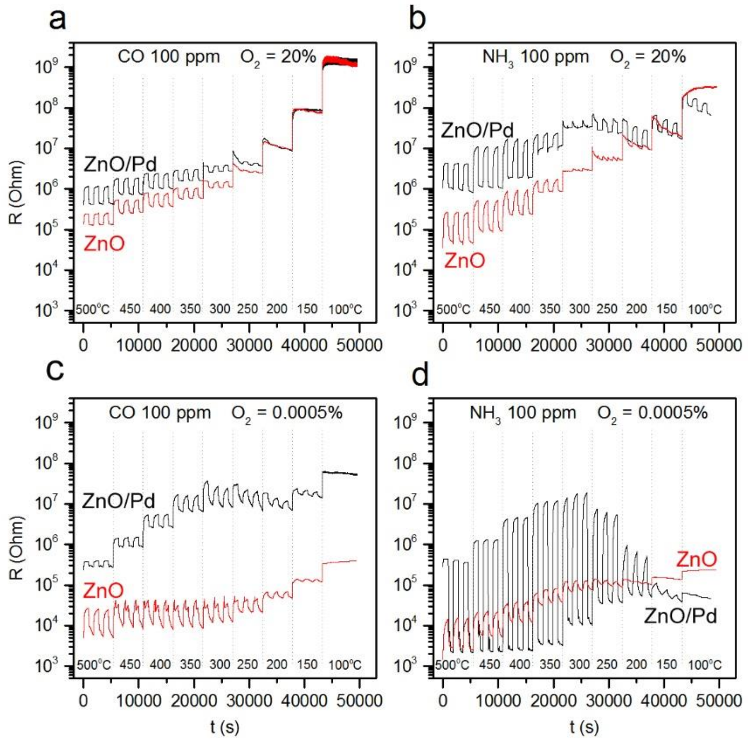

Figure S1. Transient sensor response of ZnO and ZnO/Pd nanofibers to 100 ppm of reducing gases CO **(a,c)**, NH_3 **(b,d)** in atmosphere with different oxygen backgrounds of 20% **(a,b)** and 0.0005% **(c,d)** in the temperature range 100 – 500°C.

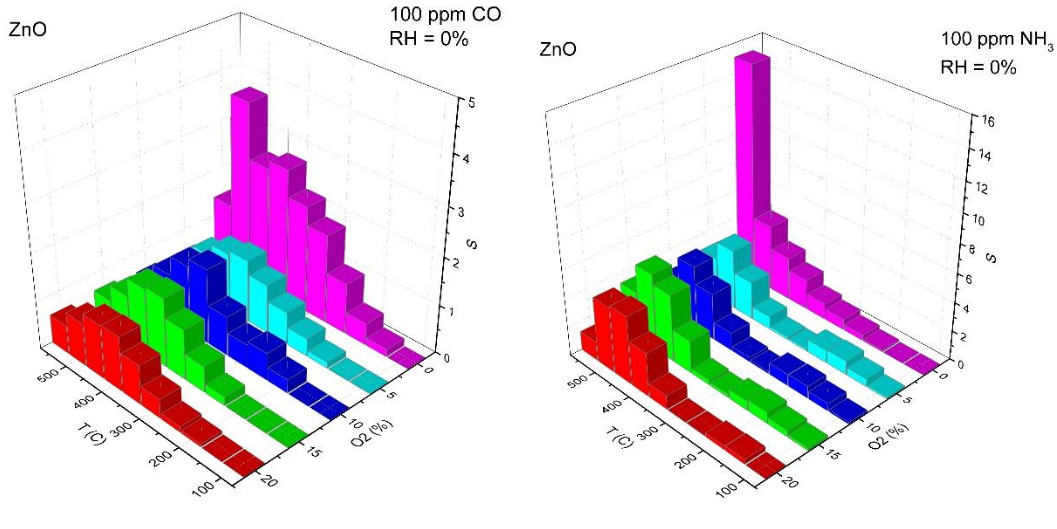

(a) **(b)**

Figure S2. Temperature dependences of sensor response of ZnO nanofibers to 100 ppm of reducing gases CO **(a)**, NH_3 **(b)** in atmosphere with different oxygen backgrounds C(O_2) = 20, 15, 10, 5 and 0.0005%.

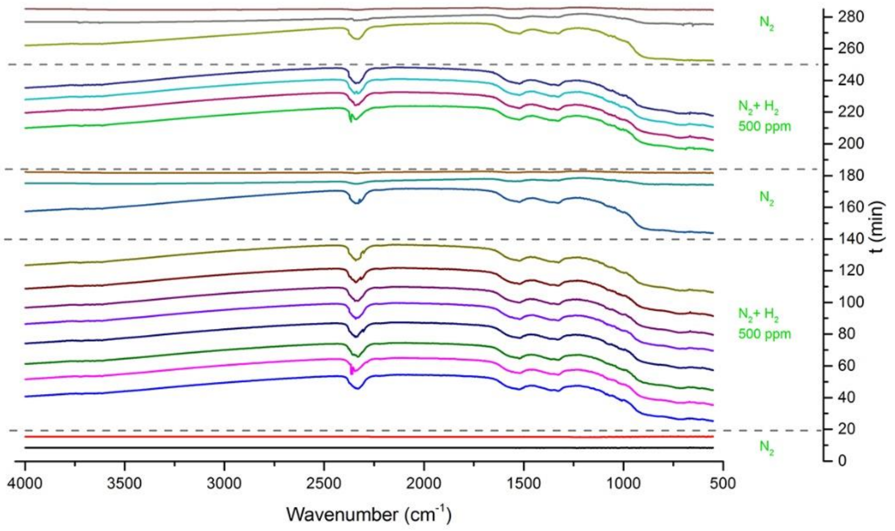

(a)

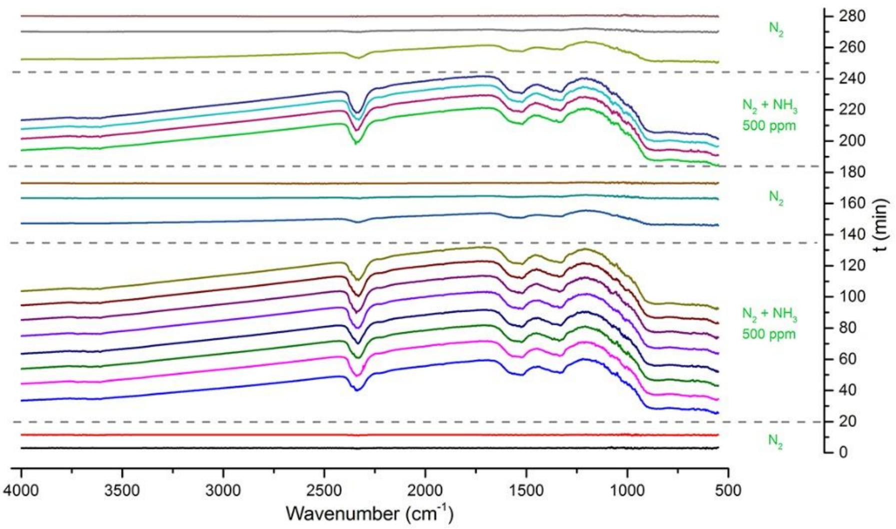

(b)

Figure S3. DRIFT spectra of ZnO/Pd nanofibers recorded in the presence of 500 ppm H_2 **(a)** and 500 ppm NH_3 **(b)** in background gas with 0.0005% O_2 at 350°C.
